# The Role of Hippo/YAP Signaling in Alveolar Repair and Pulmonary Fibrosis

**DOI:** 10.3389/fmed.2021.752316

**Published:** 2021-10-04

**Authors:** Jason J. Gokey, Saawan D. Patel, Jonathan A. Kropski

**Affiliations:** ^1^Division of Allergy, Pulmonary and Critical Care Medicine, Department of Medicine, Vanderbilt University Medical Center, Nashville, TN, United States; ^2^Department of Cell and Developmental Biology, Vanderbilt University, Nashville, TN, United States; ^3^Department of Veterans Affairs Medical Center, Nashville, TN, United States

**Keywords:** pulmonary fibrosis (PF), idiopathic pulmonary fibrosis, Hippo YAP/TAZ, review, alveolar repair, alveolar epithelial cell, fibroblast activation

## Abstract

Pulmonary fibrosis is characterized by loss of normal alveoli, accumulation of pathologic activated fibroblasts, and exuberant extracellular matrix deposition that over time can lead to progressive loss of respiratory function and death. This loss of respiratory function is associated with the loss of alveolar type 1 cells (AT1) that play a crucial role in gas exchange and the depletion of the alveolar type 2 cells (AT2) that act as progenitor cells to regenerate the AT1 and AT2 cell populations during repair. Understanding the mechanisms that regulate normal alveolar repair and those associated with pathologic repair is essential to identify potential therapeutic targets to treat or delay progression of fibrotic diseases. The Hippo/YAP developmental signaling pathway has been implicated as a regulator of normal alveolar development and repair. In idiopathic pulmonary fibrosis, aberrant activation of YAP/TAZ has been demonstrated in both the alveolar epithelium and activated fibroblasts associated with increased fibrotic remodeling, and there is emerging interest in this pathway as a target for antifibrotic therapies. In this review, we summarize current evidence as to the role of the Hippo-YAP/TAZ pathway in alveolar development, homeostasis, and repair, and highlight key questions that must be resolved to determine effective strategies to modulate YAP/TAZ signaling to prevent progressive pulmonary fibrosis and enhance adaptive alveolar repair.

## Introduction

Interstitial lung diseases (ILDs) are characterized by progressive loss of respiratory function leading to breathlessness, hypoxemia, and in many cases, death due to respiratory failure (1). Respiratory failure results from the loss of functional alveoli to participate in gas exchange and their replacement by a pathologic accumulation of extracellular matrix. Idiopathic Pulmonary Fibrosis (IPF), the most common of the idiopathic ILDs, is a rapidly progressing disorder of older adults with diagnosis typically occurring between the 60-70th year (2, 3) and mean survival of 3-5 years after diagnosis. While pirfenidone and nintedanib have been demonstrated to slow lung function decline and are approved for the treatment of IPF, large impacts on patient survival and quality of life are yet to be achieved by any IPF therapy (4, 5). Informed by human genetic studies and mouse models, the prevailing hypothesis suggests that IPF is initiated by chronic alveolar injury and failure of normal, adaptive repair mechanisms (6–10). Consistent with this hypothesis, several pathways involved in lung development have been implicated in normal lung regenerative processes including Wingless (Wnt), mammalian target of rapamycin (mTOR), transforming growth-factor beta (TGFβ), and the Hippo/Yes-associated protein (YAP) (11–14) pathway. Emerging data indicate that the Hippo/Yap pathway plays a central and essential role in normal lung repair, and dysregulation of this pathway is a prominent feature of pathologic lung repair and pulmonary fibrosis.

## The Hippo/Yap Pathway

The Hippo/YAP pathway consists of the core Hippo components, the mammalian Sterile20-like 1 and 2 kinases (MST1/MST2), also known as Serine-Threonine kinases 4 and 3 (STK4/STK3), respectively, interact with Salvador (SAV1) to phosphorylate large tumor suppressor kinases LATS1/2, that in turn phosphorylates YAP and its homolog TAZ to prevent nuclear localization. In the absence of this phosphorylation cascade, YAP/TAZ localizes to the nucleus where it complexes with one or more of a repertoire of binding partners, most prominently the TEADs 1-4, to regulate transcription of genes associated with cell migration and proliferation (13, 15, 16). YAP/TAZ signaling has increasingly been linked to regulating cell behavior both independent of and in coordination with other developmental pathways (15). In MCF10A breast cancer cells, YAP interacts with the transcription factor KLF5 to regulate cell proliferation (17). YAP has also been demonstrated to influence signaling through several pathways that regulate epithelial cell proliferation. Moreover, YAP has been shown to inhibit phosphatase and tensin homolog (PTEN), which itself normally inhibits the mTOR/Pi3K pathway (18). Similarly, mTOR activity impaired the normal 14-3-3 ubiquitin regulated autophagic degradation of YAP, thereby accumulating nuclear YAP and increasing YAP activity in the kidneys of tuberous sclerosis complex model mice (19). Wnt signaling is well-recognized to regulate progenitor cell fate, and YAP and Wnt signaling have been shown to activate and antagonize the other pathway in different contexts regarding cell fate decisions. The mechanism underlying these context-dependent divergent regulatory roles are not yet well-understood (20–23). In breast and colon cancer cells, as well as in skin fibroblasts, YAP/TAZ and SMAD, a core component of TGFβ signaling, form complexes to direct cell transcriptional activity to direct proliferation and differentiation (24–26). To further complicate the role of YAP, recent findings in the heart reveal that YAP/TAZ regulate chromatin accessibility to regulate cardiomyocyte differentiation (27). These pathways are all activated during development of the lung epithelium and abnormally regulated during fibrosis. It is unlikely that these pathways act independent of each other; therefore, understanding the complex interactions between them may be essential in determining treatment options for fibrosis (28).

## Yap in Lung Epithelium

During lung development, Yap is required for normal airway branching (29–31), and available data suggest that precise spatial and temporal regulation of Yap activity in the airway epithelium is critical for normal development. For example, persistent activation of Yap in airway basal cells leads to hyperplasia and impaired terminal differentiation. In contrast, Yap deletion results in accelerated terminal differentiation and depletion of progenitor cells (29, 30, 32). Together, these data indicate that in the airway epithelium, Yap activity is dynamically regulated to maintain homeostasis.

Recent findings in the mouse lung have provided additional insight into the normal function of Yap signaling in alveolar development and homeostasis. Using the pan-lung epithelial-specific Sonic hedgehog cre-recombinase (*Shh-Cre*) to activate Yap *via* deletion of *Lats1/2* starting around E9.5, Nantie and colleagues demonstrated multiple striking defects in lung development, including markedly reduced lung size, failure of distal branching, near complete absence of Spc-expressing AT2 cells by E18.5, and near-complete effacement of the distal lung epithelium with Hopx+ cells; unexpectedly, Hopx+ cells were detectable as early as E11.5, suggesting that persist activation of Yap signaling the developing lung epithelium promotes abnormal and ectopic differentiation. In addition, conditional deletion of *Yap* and *Taz* using an *Sftpc-rtTA/tetO*-Cre system starting at E16.5 resulted in few Hopx+ cells by E17.5, while conditional expression of a constitutively active Yap using the same system resulted in an increased number of Hopx+ cells prior to birth (33). Together, these data indicated that Yap signaling is likely a critical factor in AT1 cell fate-commitment in the developing lung.

Subsequent studies revealed that Yap interactions explain an unresolved paradox in alveolar development. It has long been recognized that the transcription factor Nkx2-1 is necessary for lung epithelial fate specification, but puzzlingly, Nkx2-1 activation has been observed to promote both AT2 and AT1 cell differentiation. Profiling chromatin accessibility in lineage-labeled AT1 cells (using a *Wnt3a*-*Cre*) and AT2 cells (using the tamoxifen inducible *Sftpc-CreER*), Little and colleagues identified distinct regions of open chromatin containing Nkx2-1 motifs; in AT1 cells, these motifs were enriched for Tead sites. As Teads complex with Yap and/or Taz, this suggested Yap/Taz regulation of Nkx2-1 accessible sites to facilitate AT1 differentiation. Deletion of *Yap*/*Taz* in AT1 cells using a *Wnt3a-Cre* led to a shift in Nkx2-1 accessible sites which more closely resembled those seen in AT2 cells, implying unexpected plasticity of AT1 cells in the absence of active *Yap*/*Taz* (34).

Similar findings have been observed in the context of neonatal injury repair. In the neonatal period, hyperoxic lung injury leads to loss of AT1 cells, and using this model, Penkala and colleagues recently demonstrated that deletion of *Yap*/*Taz* using a *Hopx-CreER* administering tamoxifen shortly after birth, led to an increased number of AT2 cells. Using an elegant strategy combining lineage labeling of *Sftpc*+ cells with doxycycline-inducible expression of constitutively active Yap, they observed cuboidal appearing cells co-expressing Hopx and Sftpc (35). Consistent with both reports, our group has recently shown that deletion of Yap in postnatal *Sftpc*+ AT2 cells resulted in increased expression of mature AT2 cell markers including *Napsa, Sftpb*, and *Abca3*. Yap activation, induced by deletion of Mst1/2 using the *Sftpc-CreER* at postnatal day 3, opened broad regions of chromatin associated with promoters of genes involved with alveolar epithelial cell differentiation by postnatal day 14. Analysis of these opened promoter regions demonstrated enrichment for Nkx2-1, Nfib, Klf and Tead binding sites. Studies assessing the activity of the AT1 marker *AGER* demonstrated these transcription factors interact with YAP to increase expression of *AGER*. YAP activation also increased numbers of Hopx+ cells and induced the presence of cells expressing both AT1 and AT2 cell markers. Further, a subset of Yap-activated AT2 cells express markers normally associated with conducting airway epithelial cells (36), a phenotype consistent with incomplete differentiation of a subset of AT2 cells reminiscent of those found in the IPF lung. Together, these studies provide compelling evidence that Yap activation is an early and essential event in normal AT1 cell maturation and is required for maintenance of the AT1 state, but alone is not sufficient to drive complete AT1 maturation. Further, persistent Yap activation in AT2 cells which fail to complete AT1 differentiation is associated with abnormal differentiation of AECs toward an airway-like phenotype.

More recently, it has become clear that YAP signaling also plays a central role in normal alveolar epithelial development and pathologic alveolar repair. Initial clues linking YAP to the alveolar epithelium emerged from early single-cell RNA-sequencing studies of IPF and control lungs. Comparing the single-cell transcriptomes of HTII-280^+^ cells from IPF and donor lungs, pathway enrichment analyses identified upregulation of YAP target genes in IPF alveolar epithelial cells (AECs), suggesting increased YAP transcriptional activity these cells (37). Immunofluorescence analysis showed the presence of nuclear YAP and AJUBA with a loss of MST1/2 in IPF AT2 cells, suggesting that in advanced disease, there is abnormal and persistent YAP activation in AECs. The biological role of YAP in the alveolar epithelium was not clear; however, pathway analyses predicted that YAP interactions with Wnt and mTOR signaling (38). Additionally, the mechanisms leading to persistent YAP activation in the IPF epithelium have not yet been well-established. Recent data indicate epithelial barrier dysfunction may play a role. In the adult mouse lung, Zhou and colleagues demonstrated that deletion of the junctional component claudin-18 (Cldn18) destabilized Yap interactions with the Lats kinases, promoted nuclear Yap accumulation (39).

There has been somewhat less work investigating the role of Hippo/Yap signaling in repair of the injured alveolus during adulthood. In a bacterial pneumonia injury model, LaCanna and colleagues demonstrated that Spc^+^ cells underwent proliferation and differentiation, which correlated with increased Yap/Taz following lung injury. Deletion of Yap/Taz in these cells resulted in prolonged inflammatory response and delayed alveolar repair (12). This work also raises the possibility that Yap may play a role in lung immune response, another process that is dysregulated in the IPF lung. Deletion of Taz in adult mouse AT2 cells resulted in reduced AT1 cells differentiation in organoids and bleomycin induced lung injury (40). In the Hermansky Pudlak Syndrome type 2 *pearl* mouse model that is associated with markedly increased susceptibility to experimental fibrosis, the Yap target Ajuba was increased in nuclei of AT2 cells, which correlated with increased AT2 cell proliferation (41). These findings, along with those in the embryonic and postnatal lung, suggest that Yap/Taz regulation may be biphasic following injury, with an initial increase in Yap activity that increases AT2 cell proliferation, followed by downregulation of Yap/Taz in AT2 cells to maintain the AT2 cell population. Understanding Yap/Taz dynamics following injury, in both the epithelium and in other cell types, will be essential to developing strategies to target the pathway for treatment.

## Yap Activity in the Lung Fibroblasts

While abnormal epithelial remodeling is a hallmark of IPF, fibroblasts are the primary effector cells producing the pathologic extracellular matrix that accumulates in disease. Increasing evidence highlights a role of YAP/TAZ signaling in fibroblast activation, and several studies have elucidated upstream regulation and downstream effects of YAP/TAZ in the context of lung fibrosis.

Initial findings by Liu and colleagues found that YAP/TAZ were increased in the fibroblasts of IPF fibroblasts in regions of fibrosis, whereas YAP/TAZ nuclear localization was largely absent in the normal lung. Further analysis demonstrated that IMR-90 fibroblasts cultured on matrices of various stiffness revealed that nuclear YAP/TAZ increased on stiffer matrix. In these studies, YAP/TAZ regulates TGFβ signaling through *SERPINE1* and plasminogen activator inhibitor 1 and was associated with enhanced fibroblast activation. These data indicated that YAP/TAZ signaling is central to matrix-stiffness regulation of fibroblast activation, and highlighted YAP/TAZ as a potential fibroblast therapeutic target (42). In follow-up studies, this group unexpectedly observed exacerbation of bleomycin-induced lung fibrosis when siRNA was administered intranasally to globally inhibit Yap/Taz in the lung (43), suggesting a more nuanced approach, either temporally or cell-type specific, to targeting this pathway will be required.

To this end, several studies have shed further light on the signaling mechanisms that mediate Yap/Taz activation in fibroblasts. G-protein-couple receptors have been shown to regulate Yap activity in response to TGFβ stimulation (44). In the bleomycin injury model, the profibrotic effects of Yap have been shown to be regulated by TGFβ *via* sphingosine-1-phosphate (S1p), a signaling lipid, and use of an antibody blocking S1p receptors reduced TGFβ-mediated YAP activation (45). Aravamudhan et al. (46) showed that the TGFβ related protein tank binding protein kinase-1 (TBK1), is regulated by mechanosensing of the lung fibroblast and is upstream of YAP activation. TBK1 was an attractive target as there are readily available drugs to target it and it may provide a potential way to specifically regulate YAP induced fibroblasts activation associated with progression of pulmonary fibrosis. Several findings by Sudhadevi et al. point to how changes in the extracellular matrix can be directly linked to the effects of YAP/TAZ. Constant expression of YAP/TAZ proteins in immortalized fibroblasts lead to progressive hardening of matrices and eventual fibrosis when transferred into murine lungs, thereby indicating that YAP/TAZ localization to nuclei can confer fibrogenic potential to fibroblasts. Surprisingly, IPF-derived fibroblasts and normal lung-derived fibroblasts had virtually indistinguishable levels of YAP/TAZ expression and could reverse localization when cultured on soft matrices. This suggests that either type of fibroblast retains the ability to respond to their mechanical environment (42, 45). Altogether, these findings imply that the dynamics of YAP/TAZ activity in lung fibroblasts are more dependent on environment than previously known. Understanding how to target YAP/TAZ upstream of fibroblast activation may develop ways to delay disease progression. Recent work used a high throughput screen of YAP activated human primary fibroblasts to identify that HMG-CoA reductase inhibitors (commonly referred to as “statins”) inhibit nuclear YAP localization and that simvastatin treatment reduced nuclear YAP and reduced fibroblast activation markers in bleomycin induced lung fibrosis mouse models (47).

The profibrotic mechanisms downstream of Yap have been investigated to a somewhat lesser degree. It has been shown that once Yap is activated in the fibroblasts, it upregulates the transcription of Twist1, which interacts with Tead-1 to induce profibrotic expression of collagen-1, fibronectin, and connective-tissue growth factor (Ctgf). In this same study, miR-15a, a microRNA that is downregulated in IPF patients, is shown to have potential in preventing fibrogenesis by downregulating YAP expression (48). Noguchi et al. (49) found that TAZ was specifically upregulated in IPF fibroblasts, and utilizing HFL-1 human fibroblasts found that TAZ specifically upregulate myofibroblast phenotypes including increased expression of alpha-smooth muscle actin (αSMA) in concert with activation of CTGF. Together, this body of work builds a strong case that modulating YAP activity offers promise for ameliorating pathologic fibroblast activation and interrupting a feed-forward cycle wherein fibrosis activates YAP which begets further fibrosis (50).

## Future Work and Open Questions

**Figure 1:**
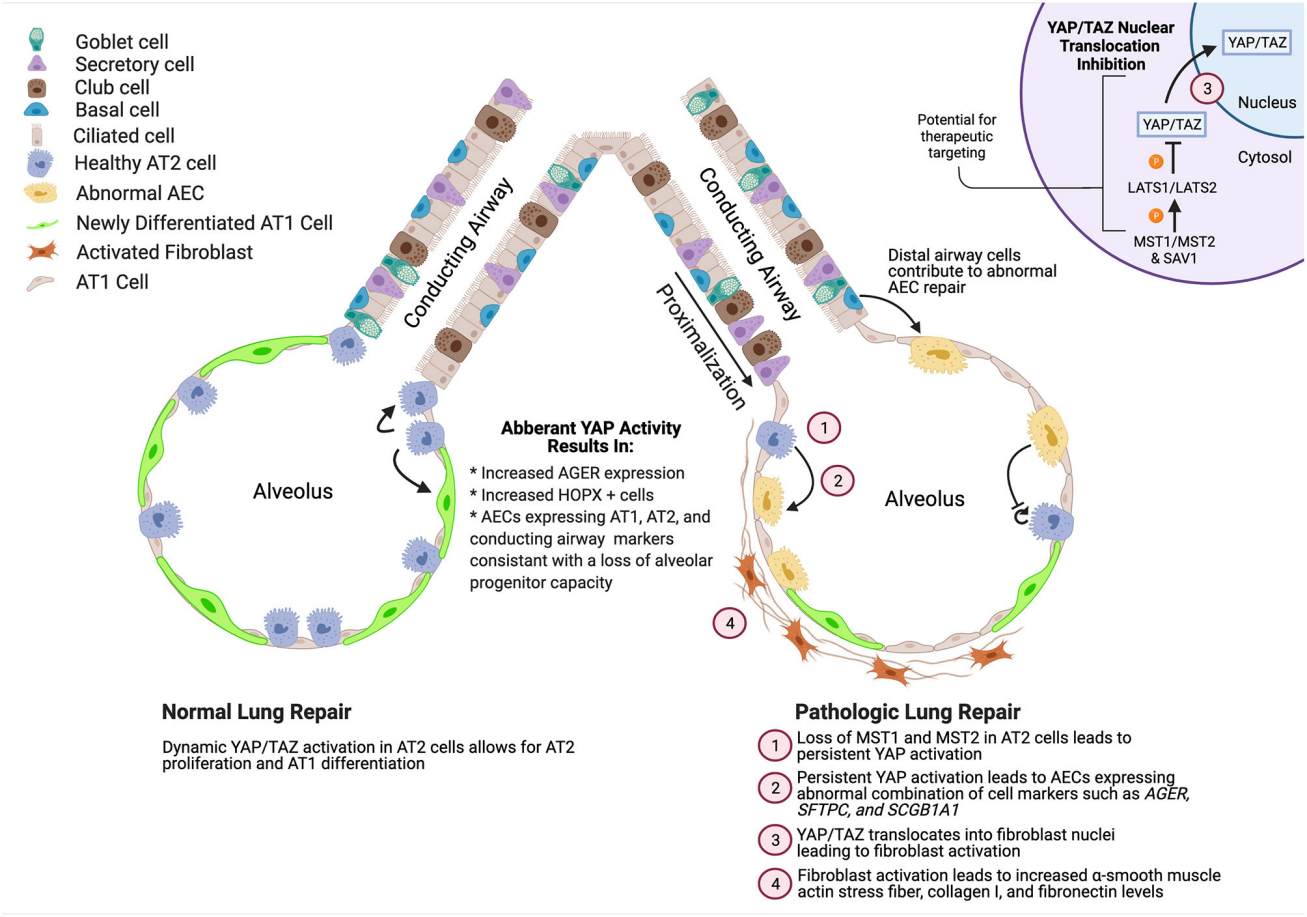
Summary of role of YAP activity during normal and pathologic alveolar repair. YAP/TAZ activation during normal development enhances AT2 cell proliferation and promotes AT1 cell differentiation while deletion of YAP/TAZ leads to increased expression of mature AT2 cell markers. During repair, YAP/TAZ is initially activated, followed by a decrease in activity. However, in ILDs such as IPF, YAP/TAZ is aberrantly activated leading to abnormal differentiation of AECs and activation of fibroblasts. Created using BioRender.com.

With increasing understanding of the complex roles YAP/TAZ play in lung development, homeostasis, injury repair, and pathologic fibrosis, it has become clear that approaching this pathway in a therapeutic context will not be as simple as global inhibition or activation, in contrast to other organs (51). While aberrant YAP activation in the epithelium and mesenchyme contribute to failure of repair, initial YAP activation in these cells is likely beneficial for proliferation of progenitor populations and signaling to immune cells (12). Several studies have found that immune cell regulation plays a role in IPF pathogenesis which are reviewed elsewhere (52–54), and defining the role of YAP in immune response during lung injury may shed further light onto the role of YAP in lung repair. Likewise, the lung endothelium has been shown to recruit immune cells and endothelial cells are aberrantly regulated in bleomycin induced lung injury contributing to fibrotic repair (55, 56). YAP activation in the endothelium was recently found to be beneficial in protecting the lung from ventilator induced lung injury, another form of lung injury that involves multiple cell types (57). The recognition that YAP/TAZ are dynamically regulated and active in multiple cell types makes detailed, nuanced, context-specific understanding of the components that regulate this pathway essential for targeting it for effective therapeutic targeting (Figure 1). The concept that altered tight junction activates YAP in AECs, combined with increased matrix stiffness associated with increased collagen deposition induces YAP in fibroblasts, and MST1/2-the inhibitors of YAP nuclear localization are decreased in IPF AECs cells, indicates a system in which YAP/TAZ is aberrantly activated in both the epithelium and mesenchyme. Designing a system to attenuate YAP activity, without completely blocking the apparent requirement of YAP to regenerate the AT1 cells, will require a deeper understanding of the YAP dynamics and epithelial/mesenchymal cell interactions. Emerging organoid coculture systems hold promise for careful mechanisms studies addressing these questions (14, 58–63). While it seems likely that innovative cell-type targeting and/or administration strategies may be required, pharmacologic therapies targeting the YAP/TAZ pathway have intriguing potential to prevent/reverse lung fibrosis and promote adaptive lung repair.

## Author Contributions

JG, SP, and JK performed literature review, wrote, and edited the manuscript. All authors contributed to the article and approved the submitted version.

## Funding

This work was funded by NIH R01HL145372, R01HL153246, and the Pulmonary Fibrosis Foundation Scholars program with funding from Boehringer Ingelheim Pharmaceuticals, Inc. The funder was not involved in the study design, collection, analysis, interpretation of data, the writing of this article or the decision to submit it for publication.

## Conflict of Interest

The authors declare that the research was conducted in the absence of any commercial or financial relationships that could be construed as a potential conflict of interest.

## Publisher's Note

All claims expressed in this article are solely those of the authors and do not necessarily represent those of their affiliated organizations, or those of the publisher, the editors and the reviewers. Any product that may be evaluated in this article, or claim that may be made by its manufacturer, is not guaranteed or endorsed by the publisher.
